# The problem of water body status misclassification—a Hierarchical Approach

**DOI:** 10.1007/s10661-018-6603-9

**Published:** 2018-04-03

**Authors:** Małgorzata Loga, Anna Wierzchołowska-Dziedzic, Andrzej Martyszunis

**Affiliations:** 1 Faculty of Building Services, Hydro and Environmental Engineering, Warsaw University of Technology, Nowowiejska 20, 00-653 Warsaw, Poland; 2 The Polish National Energy Conservation Agency, Al. Jerozolimskie 65/79, 00-697 Warsaw, Poland; 3 Graduate Faculty of Building Services, Hydro and Environmental Engineering, Warsaw University of Technology, Nowowiejska 20, 00-653 Warsaw, Poland

**Keywords:** Water body status, Uncertainty measures, Probability of misclassification, Surface water monitoring classification

## Abstract

This article addresses the issue of estimating probability of misclassification (PoM), when assessing the status of a water body (w.b.). The standard deviation of a monitoring data is considered a good measure of the uncertainty of the assessed w.b. status. However, when PoM is to be estimated from the biological data, a problem caused by too few monitoring data emerges. The problem is overcome by developing Monte-Carlo models to simulate sufficient synthetic measurements of these elements, thereby accounting for random “disturbances” in the measurements. At each level of a procedure, called the Hierarchical Approach, values of PoM were derived from the Monte-Carlo-simulated data as for the assessment of w.b. status. It is assumed in the Hierarchical Approach that PoMs on each upper level can be estimated by processing PoMs inherited from the lower levels. Data from the river monitoring systems in three Polish regions were used in the study. Values of PoM calculated for biological elements show that 70–80% of cases belong to < 0.0, 0.1 > interval, whereas PoMs for physico-chemical elements in only 20% belong in this interval whereas for 25–40% of cases, PoMs are greater than 0.5. Moreover, when analyzing PoMs for cases when the w.b. status was classified as good, 22–52% of them are characterized by 0.5 or higher probability to be assessed wrongly. These pessimistic results suggest the need for formulation of new directions for future research in determining the PoM (in general, the uncertainty) of the w.b. status estimated from monitoring data.

## Introduction

To ensure that, ultimately, water resources on their territories reach a *good* status, all the European Member States have adopted the Water Framework Directive (WFD) as the common setting for their water policies. This manifested itself in taking up the legally binding obligation of—preparing and implementing the River Basin Management Plans (RBMP). These documents define the 6-year periodic activities focused on improvement of water quality in the country. The elementary subjects of RBMP are water body (w.b.), the unit onto which water resources have been partitioned. Each RBMP consists, in particular, of assessing the state of water bodies based on water monitoring data and suggesting undertaking suitable corrective actions where the assessed state of w.b. does not (or, likely, will not) satisfy the water quality standards. If necessary, RBMP may include also a revision of the country’s current water monitoring system—its structure and functionalities. To this end, the water monitoring systems in the EU countries have been set into operation in accordance with the WFD guidelines. Depending on the country water situation, specific infrastructure and procedures for monitoring of its water resources have been established in each country. The key role is played by methods that enable assessment of the status of all the water bodies using water monitoring data.

This article addresses two procedural steps relevant for WFD-guided water management—assessment of a water body status from the water quality measurement data and estimating uncertainty of the assessed status. The multistep procedure of a water body classification is precisely described in the WFD guidelines and has been successfully implemented in the national legislations. However, the outcomes of the described classification procedure do exhibit certain degree of uncertainty and can be, therefore, erroneous in some instances. The uncertainty of the assessed w.b. status is a complex issue because it is generated by a long chain of consecutive stochastic processes—starting from the environmental and man-related causes induced by external sources in the catchment and the random dynamics of aquatic environment itself, followed by random nature of water sampling procedures and noise-effecting instruments used in the field measurements, through incidental disturbances of water sample transportation, to random errors inherent in the analytical methods, procedures, and measurements of water quality indices carried out in the laboratories. Sources of the randomness in variability and uncertainty of the water quality monitoring measurements have been discussed in detail by many authors (Clarke and Hering 2006; Gobeyn et al. 2016; Kolada et al. 2014; Kotamäki et al. 2015; Szoszkiewicz et al. 2007).

The random causes effecting the ultimate result of water monitoring procedures—the assessed water body status—inescapably make it a random variable. Using probabilistic interpretation of the w.b. classification outcomes has proven to be very fruitful. For instance, the well-known statistical notions, like all measures of scatter, can be readily applied to define the uncertainty of the assessed w.b. status (class), see for instance Kelly et al. (2009), Clarke (2013), and Carvallo et al. (2016). Probabilistic approach fits very well to the hierarchical method used for estimating the probability of water body misclassification. The method, originally proposed by Loga (2012) for physico-chemical indicators, is extended and exemplified in this article for biological indicators of water quality.

Description of spatial and temporal variability of water quality indices as well as estimates of uncertainty of the water quality measurements is obviously related to the water monitoring schemes and procedures being applied and, in particular, to the frequency of water sampling. The issue of this relationship has been also studied and addressed recently by some authors (Facchi et al. 2007; Naddeo et al. 2013; van der Grift et al. 2016).

In general, water body misclassification may result in essentially detrimental consequences in two cases:

- the assessment resulting in *good* status of water body when its true status is lower than *good*. This can restrain water authorities from implementing (otherwise necessary) corrective or remediation actions in a given water body or within its catchment,
- the assessment resulting in *bad* status of water body when the true status of w.b. is higher than *bad*. This can trigger a decision of implementing costly remediation measures while, in fact, they are not necessary.

This leads to the general, and still unanswered, question concerning future water monitoring programs “what level of uncertainty in w.b. status assessed from the measurement data gathered by the monitoring system can be accepted by the water managers when both economic and environmental criteria are applied?”

In this study, however, much simpler question is put forward—what is the probability of misclassification (PoM) of the water body status when the “standard” WFD assessment procedures are applied to water quality data routinely gathered by the river monitoring system? Series of the riverine water measurement data from three selected regions in Poland is used to answer this question.

Probabilistic approach to monitoring data together with Hierarchical Approach applied together with Monte-Carlo modeling of biological indicators is presented in chapter on “Data and methods.” Results obtained from application of hierarchical method to data from three Polish regions are then followed by discussion and conclusions on implications of this study on the water management issues.

## Data and methods

Similarly to the other EU countries, monitoring of surface waters in Poland is at present organized in three types of monitoring networks (m.n.)—the surveillance m.n., the operational m.n., and the protected areas m.n. The aims, structures, and functionalities of these three monitoring systems follow the WFD guidance (CIS 2003).

To assess the ecological status of surface waters, the Polish State Monitoring Program carries out measurements of biological elements, physico-chemical quality indicators, and concentrations of specific non-priority substances. So-called priority substances are measured to assess the chemical status of surface waters. The monitoring data are collected within the data bases and, when required, processed in the next procedural step—the assessment of water body class. Apart from the fact that there were several changes in the approach to monitoring of hydromorphological quality elements (Szczepański 2012; Szoszkiewicz et al. 2016), indicators of hydromorphological conditions are not taken into account in this analysis considering their relatively smaller influence on the w.b. status (class) assessment compared to other factors. Also, the CIS guidance specifies that analyzing hydromorphological quality elements is required for natural water bodies only in the case of fulfilling the *high* status conditions both by biological and physico-chemical quality elements. When *good* or less-than-good class is assessed from biological or physico-chemical monitoring data, hydromorphological conditions are never decisive for the ecological status assessment nor for PoM.

### Study area and measurement data

**Fig. 1:**
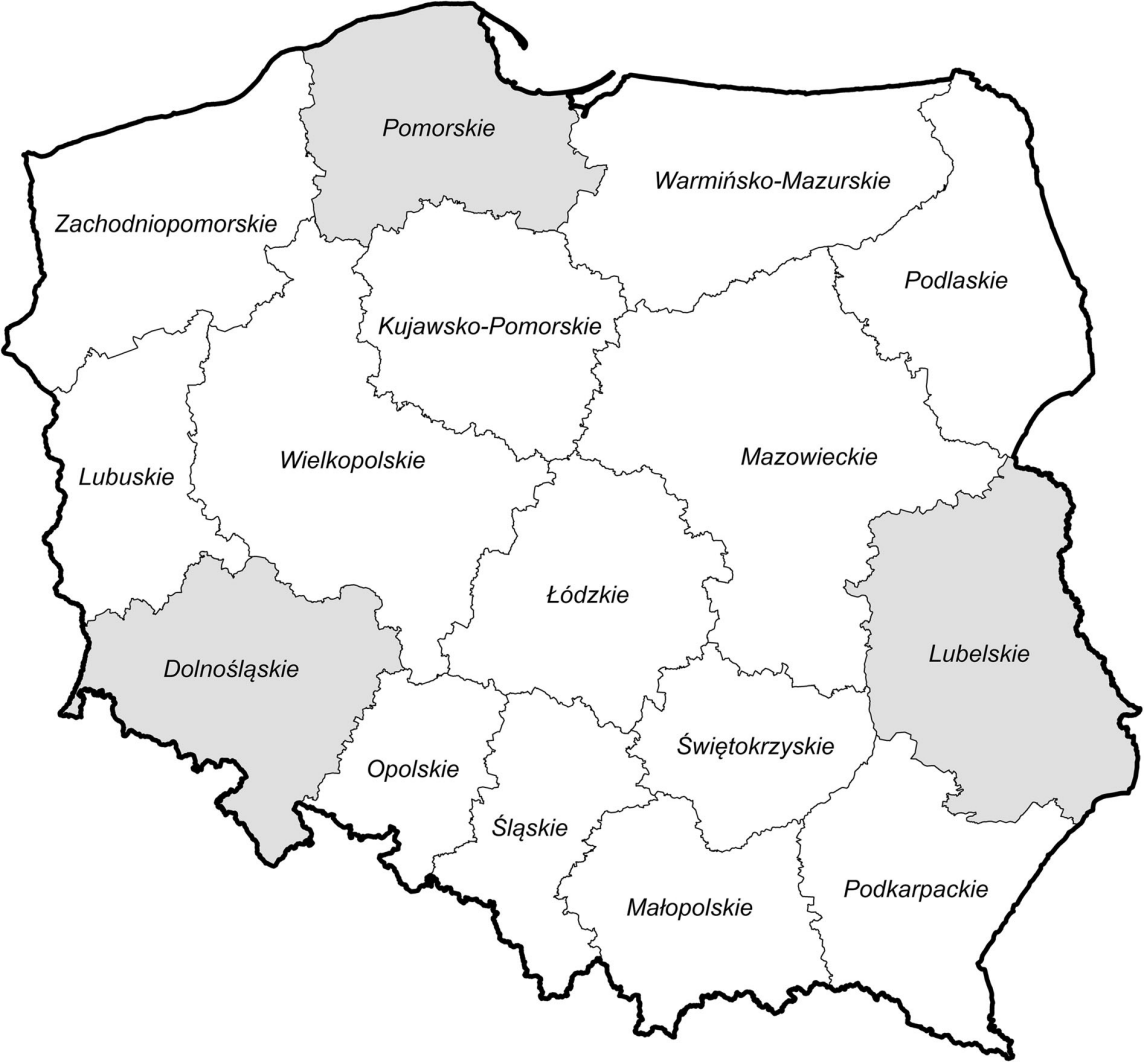
Location of the Pomorskie, Dolnośląskie (Lower Silesia), and Lubelskie regions on the territory of Poland

The basis for this study was monitoring data of all the WFD-imposed water quality indicators collected by the Voivodship Inspectorates of Environment Protection of three Polish provinces (Fig. 1)—the Dolnośląskie, the Pomorskie, and the Lubelskie Voivodships within the period 2006–2015. For the Lubelskie Voivodship, only data from the last water management cycle, i.e., from the period 2010–2015, were accessible and analyzed. The rivers of the three provinces were chosen to represent three geographical regions with distinct landscapes and substrates.

From all 26 abiotic types of Polish rivers (J. Laws 2011 No. 258, item 1549), almost all types were represented in the study, except of typical Carpathian mountain streams. Types of water bodies, which were included in the analyses, are listed in Table 1.

**Table 1:** The study regions

Voivodship	Area (km^2^)	Catchment	Type of land cover	Prevailing water body types
Dolnośląskie	19,947	Catchment of the Oder River	29.6% forest area 44% arable land	Lowland, upland, and mountain rivers
Pomorskie	18,293	Catchments of estuarial rivers flowing directly into the Baltic Sea	40% forest area 40% arable land	Sandy lowland brooks, loam lowland rivers
Lubelskie	25,155	Catchment of Vistula River (Wieprz, Bug, Bystrzyca, Huczwa, and Krzna)	23.3% forest area 70% agricultural land	Lowland sandy rivers, upland rivers, with organic substrate

Table 2 shows number of monitoring data sets within the three regions grouped according to water quality elements used for classification of water bodies status. By one data set, it is understood monitoring data from a particular w.b. collected within 1 year.

**Table 2:** Monitoring data sets used for classification of water bodies

Number of data sets used for classification of the water status	Dolnośląskie Voivodship	Pomorskie Voivodship	Lubelskie Voivodship
Monitoring of biological elements	508	368	197
Monitoring of physico-chemical elements	797	371	248
Monitoring of non-priority specific pollutants	375	266	90
Monitoring of priority substances	209	227	47

### Measures of uncertainty of water body status

Not only the status (class) of all water bodies is to be assessed in the EU countries once in 6 years but it is also required by the WFD to estimate the w.b. status uncertainty using measures like *risk*, *precision*, or *confidence*. These three measures of uncertainty have been introduced by the Guidance document No. 7 (CIS 2003).

Mathematically, a *risk* is measured as a product of probability of something happening and the measure of consequences resulting from the action taken in spite of uncertainty (e.g., costs or financial losses when the event does happen) whereas *precision* can be measured as a half of confidence interval.

In order to compare precisions of various water quality indicators, it is required to use *standardized precision*, i.e., precision divided by the mean value.

*Confidence*—similarly to the confidence interval (Montgomery and Runger 2010)—is the measure of the water body status uncertainty defined as the probability (expressed as a percentage) that in fact, the indicator value (usually the mean value) calculated from the data does lie within some range of values with specified limits.

Consistently with the water monitoring procedures adopted in Poland, also in this study, it has been assumed that the w.b. status, based on and assessed with the use of the statistical sample mean value of water quality data, represents the “true” status.

The statistical sample standard deviation (Montgomery and Runger 2010) calculated from measurement has been adopted in this paper as a measure of uncertainty (or variability) of each indicator mean. The two assumptions allow to calculate the confidence and/or precision of the status class for any water quality indicator.

The focus of this paper is on the notion of probability of misclassification (PoM), which can be considered as the measure of risk of the erroneous assessment of the water body status. The analysis is restricted to the probability of such erroneous judgment without taking into consideration its economic consequences, like financial losses. Another concept, which simplifies further analysis, is based on the plausible assumption that the probability of misclassification of ecological status of particular water body can be calculated as the probability of misclassification linked to the one calculated for the water quality element which is “responsible” for the w.b. resultant status. This idea is in line with the rule OOAO (“One Out All Out”), which specifies the element classified in the lowest class as representative for the whole set of elements and whose status labels status of the set. Such element is called also a “decisive element” as its class (status) is being inherited by the biological, physico-chemical element class or eventually ecological status.

As a hierarchy is deeply rooted in the WFD assessment procedures, this paper uses the Hierarchical Approach, mentioned before, as a basic framework for the analysis. Theoretically, the probability of misclassification of some water quality indicator—PoM—can be calculated from the following formula:

1 $$ \mathrm{PoM}=1-\underset{l(i)}{\overset{u(i)}{\int }}g\left(\overline{x}\right) dx $$

where

*l*(*i*), *u*(*i*): specified lower and upper limits of the true class “*i*” of the indicator mean value
$$ g\left(\overline{\mathrm{x}}\right) $$
distribution function for the indicator mean value.

It has been assumed in this research that the distribution function for the indicator mean value—$g\left(\overline{\mathrm{x}}\right)$—can be approximated by the normal distribution function with its mean equal to the “empirical mean” (the mean estimated from the statistical sample of measurement data) and the empirical standard deviation (the sample standard deviation estimated from the measurements data) divided by square root of number of data.

Using R (R Core 2012), PoM for each indicator class “*i*” has been calculated as the sum of probabilities that the indicator mean value has been classified by the assessment procedure to class “*i*−1” or class “*i*+1” respectively. For the given data, it was not necessary to calculate similar probabilities for more distant classes.

### Coping with uncertainty measures for biological indices

In general, without sufficiently long series of biological quality indices, there is hardly any meaningful way of estimating, on the acceptable level of confidence, value of the statistics describing random spread of biological indicators in a given water body. Still, some estimates can be chosen and, together with the corresponding statistics for the physico-chemical indices, can be subsequently used in estimating PoM of the assessed ecological status of the water body.

From all five WFD biological quality elements of riverine waters (phytoplankton, phytobenthos, macrophytes, macroinvertebrates, and fish), fish index was not analyzed in this research as it was only recently introduced to the Polish river monitoring programs. The other four biological quality elements have sufficiently long history in monitoring of the country surface waters to allow for a reliable assessment of the water body status and estimation of the w.b. status uncertainty in terms of PoM*.* In order to substitute for lacking empirical estimates of standard deviation based on the field measurements of biological indices, the Monte-Carlo (M-C) modeling approach was applied in this study to simulate “simple” field measurements of primary parameters necessary for estimation of the indices’ values.

For each of the four biological elements, the corresponding M-C model was developed and then used for performing multiple repetitions (of order 1000–10,000) of the simulated measurements (Loga and Wierzchołowska-Dziedzic 2017). The M-C random number generators were producing a multitude of realizations of normal random variables with zero mean and the assumed standard deviation representing disturbances to measured values of the water quality elements.

To each of the historical measurement datum from the biological monitoring data base, a series of the M-C-generated random numbers were added to simulate “likely disturbances” to a single measurement and thus forming a simulated spread of the real measured value. The same approach was applied to both—the field and the laboratory measurements. For instance, when assessing the River Macrophyte Index (MIR) (Szoszkiewicz et al. 2010), the area coverage corresponding to particular macrophyte species, expressed in 9-point scale, was considered the “simple” measurement. In M-C simulations, the area coverage for each species identified in the field survey has been randomly disturbed by one degree up or down the scale. By repeating the M-C-based calculations of MIR index as many times as to stabilize the value of the sample standard deviation and classifying each generated value of MIR into one of five classes (defined by the Polish monitoring regulations J. Laws 2014 item 1482), the resultant distribution of MIR values across the five classes has been established.

**Fig. 2:**
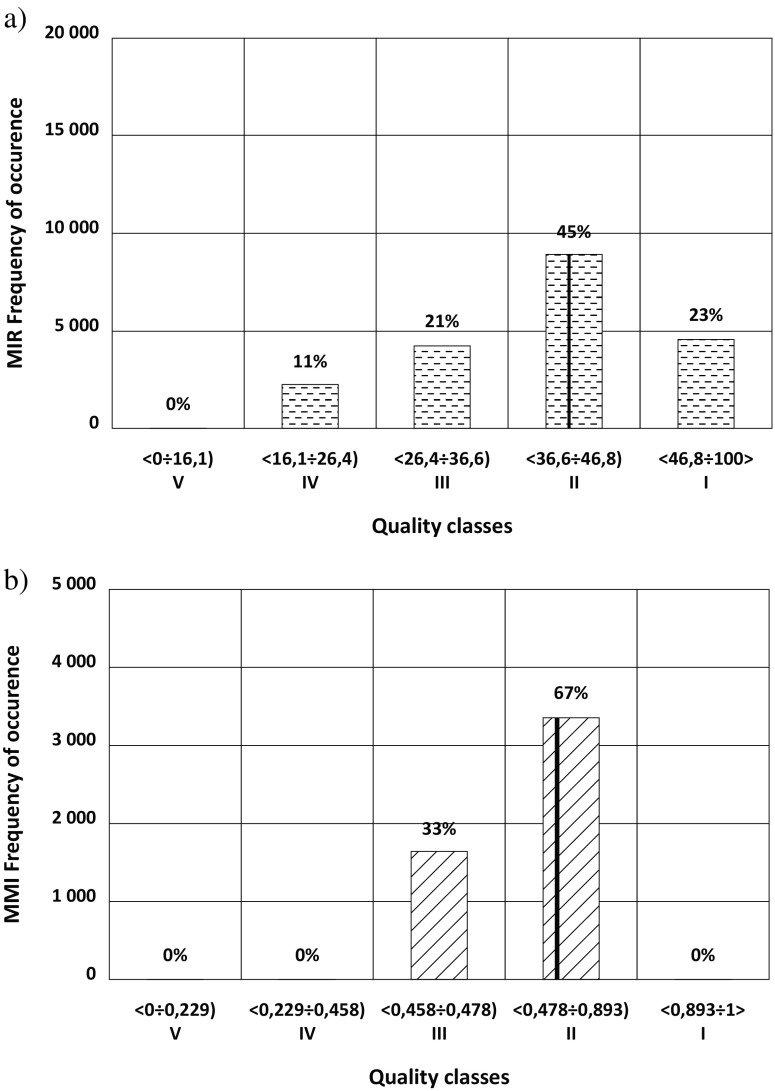
**a** Class distribution of the MIR index (Nysa Łużycka River—year 2012) resulting from random distortions by 1 point in 9-point scale of the macrophyte species cover. **b** Class distribution of MMI for 20% random error in the measured number of individuals of macrozoobenthos in samples from the Kamienica River (year 2009). The vertical line indicates the MIR and MMI values of the actual measurement in each year. The numbers above the bars specify the percentage of cases in which values of the index have fallen into particular classes (Loga and Wierzchołowska-Dziedzic 2017)

Fraction of the MIR values—calculated for the randomly disturbed *area coverage* and belonging to other classes that the class corresponding to the undisturbed MIR value—was used to calculate the probability of misclassification (PoM) from formula (1). As an example, index in 2012 together with the MIR distributions resulting from random distortions of the measurements for the Nysa Łużycka River is presented in Fig. 2a).

For phytoplankton (IFPL) index (Picińska-Fałtynowicz 2012), the concentration of every chlorophyll measurement as well as the phytoplankton taxa abundance has been disturbed with a predefined error factor.

Phytobenthos index (Błachuta and Picińska-Fałtynowicz 2010) and multimetric macroinvertebrate index MMI (Bis 2013) have been simulated with its M-C model through randomly increased or decreased number of organisms belonging to indicative species relevant to each sub index. An example of the results of M-C simulation for the assumed 20% error in the number of individuals of macroinvertebrate taxa is shown in Fig. 2b).

For all four biological element indices, the values that have fallen into other classes than the class of index calculated from the undisturbed values of directly measured parameters have been used to calculate the probability of misclassification from formula (1).

For each biological element, values of PoM have been estimated for each class separately, i.e., PoM = PoM(*i*), (*i* = 1,…, 5), and were considered the measure of uncertainty of the assessed class the element was assigned to.

### Hierarchical Approach of estimating the probability of misclassification of the water body status

The use of the Hierarchical Approach in estimating the probability of misclassification of the assessed w.b. status follows the same hierarchical principle as the w.b. class assessment itself. At each level of w.b. status assessment procedure, the corresponding PoMs are calculated for each element and then, for the group of elements specific for the given procedural level using formula (1). The ultimate PoM is assigned to given level by applying the OOAO rule.

**Fig. 3:**
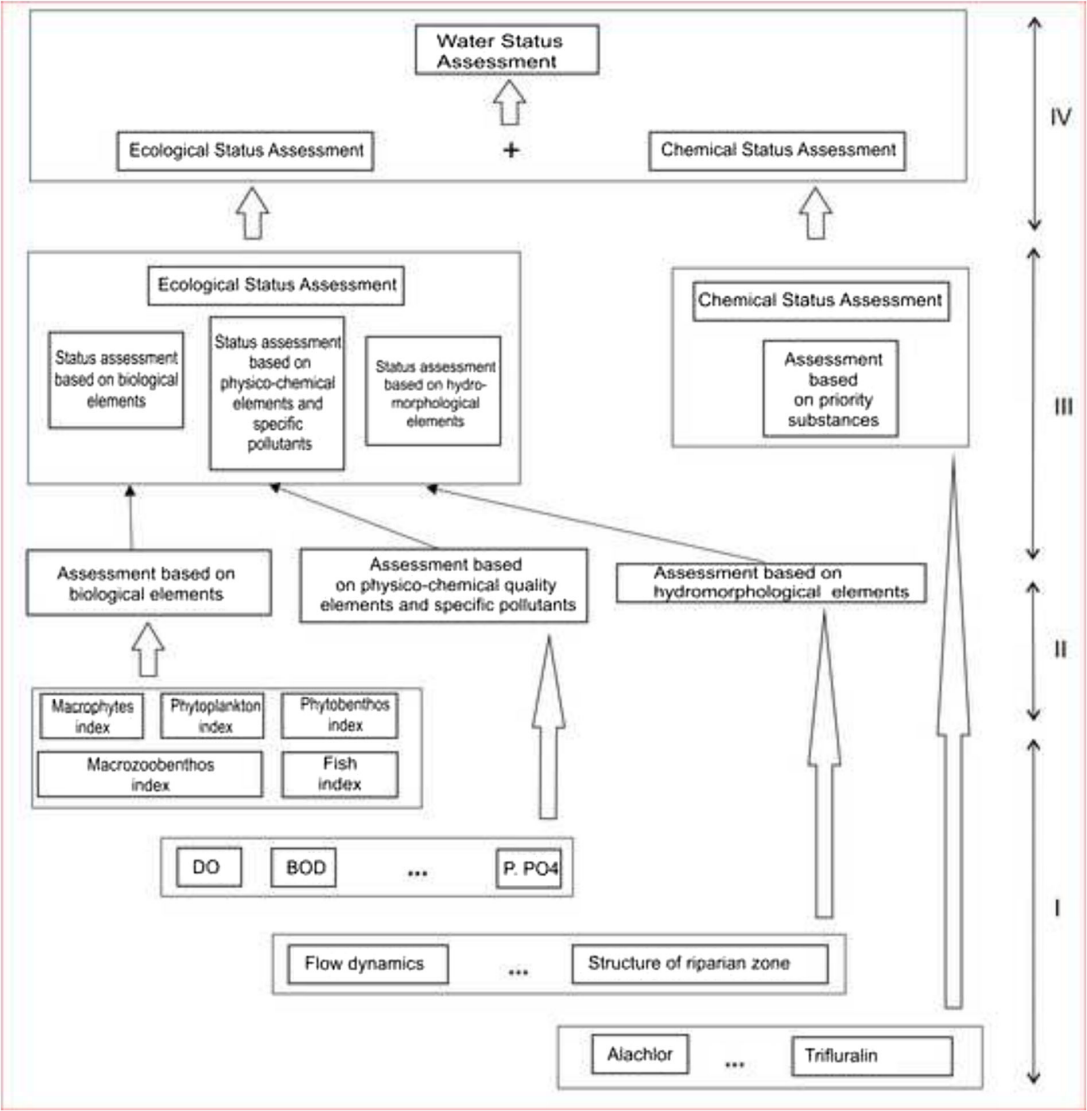
Hierarchical structure of water body status assessment (Loga 2016)

The Hierarchical Approach starts from the lowest level indicated in Fig. 3 as (level) I. For each indicator as for example macrophytes, phytoplankton, biochemical oxygen demand, and pH. (within each group of water quality elements), the class is assigned so that mean value of the indicator lies between limiting values of some class. The assessment of the indicator class is accompanied by calculation of the corresponding PoM.

The next step (level II) consists of applying the OOAO rule and selecting a “decisive indicator” from each group of water quality elements, i.e., from the biological elements, physico-chemical elements, and specific pollutants. (In the future, hydromorphological indices can be incorporated into the Hierarchical Approach as well.) PoMs for the level II classes are inherited after the PoMs of the corresponding decisive indicators from level I.

The PoM of resultant ecological status (at level III) is the one estimated for the water quality element which was decisive for the ecological status class.

If there are several water quality indicators classified equally within the same ecological status, the highest PoM value from the whole group (from all biological, physico-chemical, and hydromorphological) of water quality elements being calculated on the previous (lower) stage is assumed as the probability of misclassification for this (higher) stage, i.e. for ecological status.

Similarly to the ecological status, the PoM for chemical status (at level III) is the PoM value corresponding to the indicator (in this case for priority substance) which is decisive for the chemical status. In case there are several parameters decisive for the assessment, the highest PoM value from all probabilities of misclassification corresponding to these substances is applied.

At the highest level (indicated as level IV) of the hierarchical procedure, the resultant water body status is assessed as the worse from the two—the ecological status and the chemical status—and the resultant PoM is the highest of the two PoMs—that of the ecological and the chemical statuses.

## Results

The last two steps of the hierarchical procedure are presented in Table 3. There, the assessment outcomes of the ecological and chemical statuses of selected water bodies in the Dolnośląskie region are shown together with their PoMs. The last column contains the resultant overall classes and estimates of the corresponding PoMs for the selected water bodies.

**Table 3:** Probability of misclassification (PoM) of ecological status—examples (*SS* suspended solids, *DI* diatom index, *MIR* river macrophyte index, *PO*_*4*_ orthophosphate concentration, *N*_*org*_ organic nitrogen concentration, *DO* dissolved oxygen concentration)

WFD water body ID and corresponding river name with location in km of monitoring point from the river outlet	Class assessment based on biological elements and its probability of misclassification			Class assessment based on physico-chemical elements and its probability of misclassification			The assessed ecological status of water bodies and their corresponding probabilities of misclassification			
	Class	Classification decisive element	PoM	Class	Classification decisive element	PoM	Class	Status	Classification decisive element	PoM
PLRW6000191439 Barycz [55.9]	1	DI	0.50	2	N_org_	0.04	2	Good	N_org_	0.04
PLRW60006134489 Bielawica [9.0]	1	DI	0.00	1	SS	0.00	1	High	SS	0.00
PLRW600020163799 Bóbr [137.5]	3	DI	0.13	1	SS	0.01	3	Moderate	DI	0.13
PLRW600017146929 BystrzycaDusznicka [1]	1	DI	0.00	1	PO_4_	0.03	1	High	PO_4_	0.03
PLRW600017146929 Kanał Stawnik [1.5]	2	DI	0.00	3	DO	0.33	3	Moderate	DO	0.33
PLRW60001714549 Łacha [2.0]	2	DI	0.50	2	Ca	0.10	2	Good	DI	0.50
PLRW6000816169 Lesk [0.1]	3	DI	0.03	2	N_org_	0.07	3	Moderate	DI	0.03
PLRW60008174139 Nysa Łużycka [197.0]	3	DI	0.00	2	N_org_	0.42	3	Moderate	DI	0.00
PLRW600019133499 Oława [2.0]	3	DI	0.31	2	PO_4_	0.18	3	Moderate	DI	0.31
PLRW6000181386922 Pawłówka [0.2]	4	DI	0.06	3	DO	0.34	4	Poor	DI	0.06
PLRW60001913699 Widawa [0.5]	2	MIR	0.11	2	PO_4_	0.18	2	Good	PO_4_	0.18
PLRW60008174239 Witka [10.9]	4	MIR	0.30	1	SS	0.39	4	Poor	MIR	0.30

**Fig. 4:**
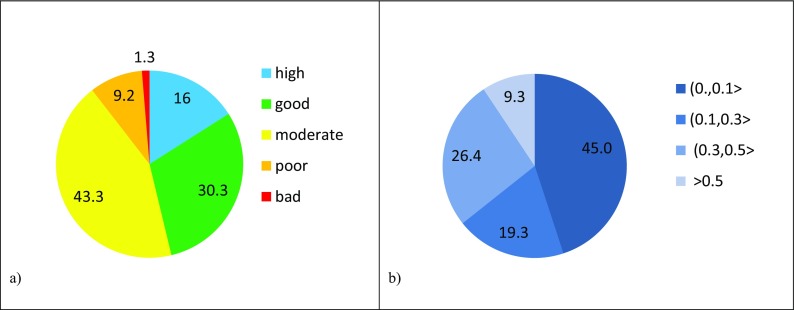
(**a**) Ecological status and (**b**) probability of misclassification of ecological status for Dolnośląskie Voivodship in the period 2006–2012

**Fig. 5:**
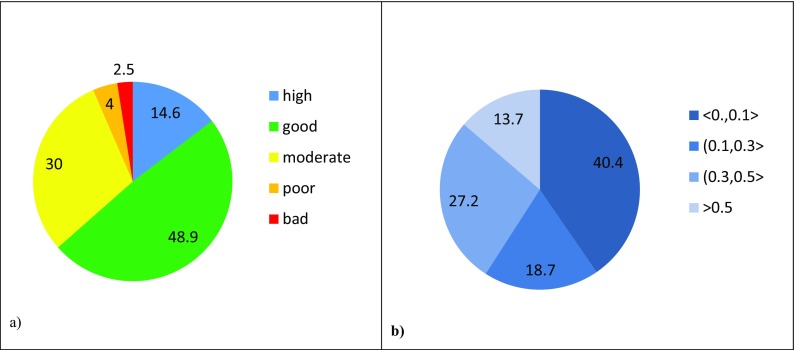
(**a**) Ecological status and (**b**) probability of misclassification of ecological status for Pomorskie Voivodship in the period 2006–2012

**Fig. 6:**
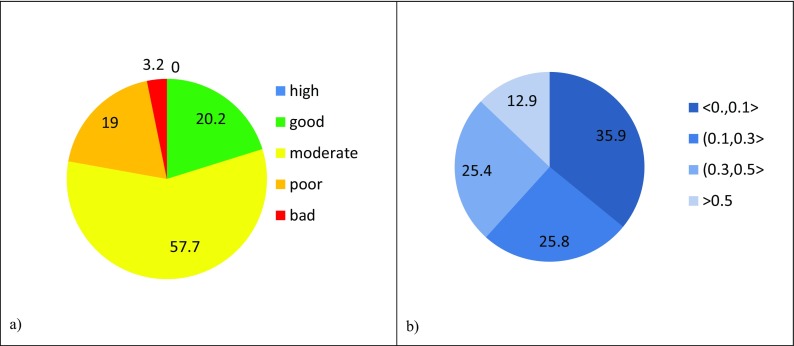
(**a**) Ecological status and (**b**) probability of misclassification of ecological status for Lubelskie Voivodship in the period 2010–2015

Classification of ecological status have been performed for all water bodies monitored on the territory of the three Polish provinces. Results of the classification are presented below in Figs. 4a, 5a, and 6a.

The largest group of w.b. in high status occurs in Dolnośląskie Voivodship. Taking into account the number of w.b.-s being at least in *good* status, it can be observed that more than 50% of such w.b.-s are in Pomorskie region. The lowest ecological status is represented by rivers in Lubelskie Voivodship where there is no w.b. in *high* status and only 20% meet *good* status conditions.

Calculated PoMs for the ecological statuses in the three regions have been grouped into four intervals: < 0.0, 0.1 >; (0.1, 0.3 >; (0.3, 0.5 >; and > 0.5. Fractions of water bodies in each of the three regions with their PoMs falling into particular intervals are quite close in their values. Pie charts of these fractions (Figs. 4b, 5b, and 6b) are therefore much alike showing that about 40% of ecological status assessments are characterized by the lowest PoM and about 10% of assessments are very doubtful as the PoM is higher than 0.5. The prevailing number (about 60%) of ecological status assessments for w.b. in all cases is within the range of probability of misclassification up to 0.3.

Noticeably, fractions of water body ecological status (Figs. 4a, 5a, and 6a) in each of the three regions do not correlate with the corresponding fractions of PoMs at all.

Calculated separately for biological, physico-chemical, and non-priority specific substances, probability of misclassification has been grouped also into four intervals. As presented in Table 4, most numerous is the interval < 0.0, 0.1 >. In the case of all the three voivodships, nearly all monitored non-priority specific substances have been classified with the smallest uncertainty of assessment, but because indicators from this group of elements rarely have been decisive for the ecological status, they were taken into consideration together with physico-chemical indicators.

**Table 4:** Probabilities of misclassification for biological, physico-chemical, and specific water quality elements and the ecological status assessment for the water bodies in Dolnośląskie, Pomorskie, and Lubelskie Voivodships (in the analyzed period of time) subdivided into the four intervals

	Dolnośląskie Voivodship			Pomorskie Voivodship			Lubelskie Voivodship		
Range of PoM	% of biological assessment with PoM within a given range	% of physic-chemical assessment with PoM within a given range	% number of ecological status assessment with PoM within a given range	% of biological assessment with PoM within a given range	% of physic-chemical assessment with PoM within a given range	% number of ecological status assessment with PoM within a given range	% of biological assessment with PoM within a given range	% of physic-chemical assessment with PoM within a given range	% number of ecological status assessment with PoM within a given range
< 0., 0.1 >	84.8	14.7	45	72	19	40.4	75.1	7.7	35.9
(0.1, 0.3 >	7.9	27.3	19.3	14.1	21	18.7	16.8	27.4	25.8
(0.3, 0.5 >	7.3	39.7	26.4	13.3	37	27.2	7.6	35.1	25.4
> 0.5	0	18.2	9.3	0.6	23	13.7	0.5	29.8	12.9

Categorization of PoMs for physico-chemical water quality elements reveals also that more than 35% of PoMs belong to interval (0.3, 0.5 >. The resultant uncertainty of ecological status (Table 4) shows that more than one quarter of the assessments can be wrong with the probability up to 0.5.

It is visible from Table 4 that the most reliable assessments are the ones based on biological quality elements. For all voivodships, more than 70% of biological assessments belong to the interval of the lowest PoM.

To see the reliability of the w.b. assessment within each class of ecological status, the four ranges of PoM are presented in Table 5 separately for each class and each voivodship. As there were only few water bodies classified in the *bad* ecological status, the last row in Table 5 that should have correspond to *bad* class is omitted. Nevertheless, the probability of misclassification for all water bodies assessed as *bad* (omitted in Table 5) was in range < 0.0, 0.1 >. It means that the *bad* status for all these water bodies is quite certain.

**Table 5:** Probability of misclassification of the assessed ecological status for all water bodies in the three regions

Ecological status	Voivodship	Percentage of water bodies in PoM classes			
		< 0.0, 0.1 >	(0.1, 0.3 >	(0.3, 0.5 >	> 0.5
High	Dolnośląskie	59.6	28.1	7.6	4.7
	Pomorskie	78.4	10.2	11.4	0
	Lubelskie	0	0	0	0
Good	Dolnośląskie	26.9	16	35.2	21.9
	Pomorskie	38.4	15.3	23.1	23.1
	Lubelskie	4	10	34	52
Moderate	Dolnośląskie	42.8	22.4	33.9	0.9
	Pomorskie	25.1	27.9	42.1	4.9
	Lubelskie	25.2	39.2	31.5	4.2
Poor	Dolnośląskie	88.8	7.1	4.1	0
	Pomorskie	45.8	16.7	29.2	8.3
	Lubelskie	91.5	6.4	2.1	0

In Table 5, very similar distribution among intervals of PoMs for both the *good* and *moderate* statuses can be noticed. It means that assessments into these two “critical” classes are characterized by high uncertainty and may possibly have costly consequences when deciding or not on undertaking corrective measures in the river catchment.

It is rather worrying to observe that in the cases of rivers in Dolnośląskie and Pomorskie Voivodships, the *good* status assessment (achievement of which is crucial from the point of view of meeting the WFD environmental objectives), only about 30% is characterized by the lowest PoM. The other 70% of the assessments of the *good* status are of higher probability to be assessed erroneously. In the case of Lubelskie region, the number of water bodies assessed as *good* but characterized by higher than 0.3 probability to be assessed erroneously equals almost 90%.

It is also worrying to see that in all voivodships, the *good* status only in 50% of cases is characterized by the lowest probability of misclassification interval. The other half of the assessment cases of the *good* status are of higher probability to be assessed wrongly. It means that, likely, quite considerable number of water bodies in the three regions assessed as being in the *good* status is assessed falsely.

Unlike for the ecological status assessment, the analogous summary for the ultimate w.b. status (and the corresponding PoMs) is not presented. The reason of refraining from such a summary was the scarcity or even the absence of measurement data of many priority substances and thus not representative character of assessments based on incomplete set of chemical indicators.

Examples of water body status in selected rivers of Dolnośląskie and Pomorskie Voivodships and the corresponding PoMs are presented in Table 6.

**Table 6:** Probability of misclassification of the status of water bodies—examples. WFD water body identification number is substituted by the river name and location of the monitoring point in kilometers from the river outlet

River and location of monitoring point in km from the river outlet	Ecological status assessment			Chemical status assessment			Overall status assessment	
	Class	Ecological status	PoM	Class	Chemical status	PoM	Status	PoM
PLRW20001929899 Wierzyca [1.6]	3	Moderate	0.00	1	Good	0.03	Bad	0.00
PLRW200019298499 Wietcisa [0.4]	3	Moderate	0.02	1	Good	0.00	Bad	0.02
PLRW60004122499 Włodzica [0.5]	3	Moderate	0.00	1	Good	0.36	Bad	0.00
PLRW6000181386922 Pawłówka [0.2]	4	Poor	0.06	1	Good	0.00	Bad	0.06
PLRW500049469 Klikawa [8.5]	1	High	0.00	1	Good	0.10	Good	0.10
PLRW60001913699 Widawa [0.5]	3	Moderate	0.21	1	Good	0.00	Bad	0.21
PLRW60008174239 Witka [10.9]	4	Poor	0.30	1	Good	0.00	Bad	0.30
PLRW60004134189 Złota Woda [5.0]	2	Good	0.00	1	Good	0.00	Good	0.00

## Discussion

The Hierarchical Approach in estimating the uncertainty of the assessed water body status was introduced by Loga (2012) and then presented in details by Loga (2016). Originally, the approach was applied to physico-chemical water quality elements as only in few cases standard deviation for biological indices could be meaningfully calculated. In the mentioned studies, only standardized precision was used as a measure of the measurement uncertainty and as the first approximation of w.b. status uncertainty. At present, when monitoring data of biological elements are more abundant and as the result of applying M-C models, the Hierarchical Approach can be extended for these elements making the issue of estimating uncertainty in water resources classification with PoM complete.

When comparing results of status assessment uncertainty expressed both by PoM and by standardized precision, no significant correlation was found. However, a general and rather obvious statement that “the smaller the sample standard deviation calculated from the measurement data, the smaller the uncertainty measure PoM” was confirmed.

Hence, by increasing the number of data in the statistical sample through increased annual frequency of water sampling, one may decrease uncertainty (precision and confidence) of the sample mean and thus lower probability that the w.b. status can be misclassified by the w.b. status assessment procedure. Choice of water sampling frequency not only is monitoring procedural issue but also has far-reaching water management consequences. In general, increasing frequency of water sampling decreases the probability of misclassification and thus reduces the risk of undertaking the corrective measures in a river or in its catchment when there is no need for that. On the other hand, too low water sampling frequency can result in small monitoring data sets and, in some instances, lead to false assessment of w.b. status as *good.* This may cause refraining by the water authorities from executing the corrective or remediation actions, which, in such a case, can induce environmental or economic losses. This is especially true as the frequency of water sampling in monitoring programs in Poland is tailored more to the available budget than to the dynamics of water quality indicators (Loga et al. 2018).

There are also aspects of w.b. status assessment especially difficult to treat mathematically within the framework of statistics, namely the various methods of aggregation (lumping) of the water quality indicators. They are introduced for the purpose of simplifying the, otherwise, complex, ecological status assessment procedures. However, by introducing discontinuities into the random functions, the aggregation methods make in many instances the statistical analysis rather a complex task.

Discussion of various methods of the indicators’ aggregation, including minimum aggregation method One-Out-All-Out (OOAO), imposed by WFD through its guidelines (CIS 2005), is presented in Boria and Rodríguez (2010), Langans et al. (2014), and Moe et al. (2015). The subject has been further analyzed by Probst (2017).

It needs to be remarked that some methods of aggregating of water quality indicators are different than the OOAO. When used within the procedure of w.b. status assessment, these methods do result in less restrictive classifications as compared to the WFD-induced OOAO rule. The methods also seem to be very appealing in Polish conditions as they create a realistic chance of increasing the assessed overall status of surface waters in the country. For, with the present use of OOAO method, as much as 76% of river water bodies and 67% of lake water bodies fail to reach *good* status (J. Laws 2016a, No 1911; J. Laws 2016b, No. 1967; Soszka et al. 2016).

It is very likely that for many water bodies in the country, their status assessed from the monitoring data does not represent the true status. When the rule OOAO is unconditionally applied to such “imperfect” measurement data—as is in this research—the biased picture of the country w.b. status emerges. The results of the misclassification analysis shown in this article clearly confirm this observation.

The calculated values of PoM confirm rather obvious observation that the higher is the value of the sample standard deviation, the higher the probability of an erroneous assignment of the class to given water quality element. In the case of Polish water monitoring procedures, the number of measurements of physico-chemical elements is usually not smaller than 12 per annum, which allows reliably estimating values of standard deviation and, in majority of cases, regarding them as acceptable measure of uncertainty for these elements. More problematic part is estimation of standard deviation for indices based on biological quality elements. Due to their slow response to exerted stresses, biological element indicators are believed to reflect the averaged characteristics of water body status within some period of time. In contrast, majority of physical processes and chemical reactions in water respond to external stresses relatively quickly and thus representing “instantaneous” state of water body. This in fact is the reason why biological elements are measured in water with much lower frequency than physico-chemical elements. Status assessment based on biological elements is performed for water body only once within each 6-year water management planning cycle, in the case of surveillance monitoring program, whereas in the case of operational monitoring program, some selected biological quality elements can be assessed in two or, rarely, in three consecutive years. However, merging the measurements of biological elements from several years into one lumped set, even in case the data are available, does not allow for determination of a stable standard deviation that could be representative for these years. This happens because of natural evolution of aquatic systems and growing pressure from human activities in the catchment. Both driving factors result in the non-stationary behavior of biological indicators making it impossible to find stationary statistical distributions for them. The contrary assumption, considering validity of the stationary approach, applied in water management (especially in view of climate warming) has been criticized in many publications (Milly et al. 2008, 2015; Kundzewicz et al. 2009). This creates a stalemate situation in statistical interpretation of biological element measurements. Some solution to this problem has been proposed in the “Data and methods” paragraph.

From the three WFD-defined uncertainty measures applicable for validating the water body status assessment—confidence, precision, and probability of misclassification—PoM seems to be the most suitable as a measure of the goodness of the assessed w.b. status. The other two measures—precision and confidence—characterize the variability of the monitoring results within the assessment period rather than contribute to confirmation or falsification of the correctness of the assessed w.b. status. In case when uncertainty interval of an indicator lies completely within the interior of a particular class of water quality, it does not influence the credibility of the assessed status. However, in the case of relatively narrow water quality class, even parameter with quite moderate spread around the measured value can contribute to false assessment of w.b. status.

The important goal of carrying out water monitoring in rivers is to check the compliance of the assessed status of the riverine water with the WFD-defined environmental goal which, for majority of water bodies, is their *good* ecological status or *good* ecological potential. As this requires a reliable assessment of the w.b. status from the monitoring data, therefore, the crucial issue is estimating PoM and using it for controlling reliability of the assessed status. Clearly, for making the water management decisions at low risk, it is desirable to assess at least *good* and *moderate* ecological statuses of water body with possibly low probability of misclassification.

It was observed that PoM is not related to class itself but rather to the decisive element related to the group. From the analysis of PoM for different groups of water quality elements studied in the three provinces, it can be concluded that PoM for biological elements in 70–80% of cases is not greater than 0.1, i.e., it belongs to < 0.0, 0.1 > interval, whereas PoM for physico-chemical elements in 25–40% of cases is greater than 0.5, and only about 14–19% of them are characterized by the lower PoM.

## Conclusions

The hierarchical method has been successfully applied to assess probability of misclassification of physico-chemical, specific non-priority, and chemical water quality elements. When supplemented with the Monte-Carlo models, simulating random disturbances in measurements of biological elements in water bodies, the method has given also reliable estimates of PoM for the assessed ecological, chemical, and overall w.b. class.

Large group of all ecological status assessments is characterized by the lowest PoM (within range up to 0.1) but the number of such w.b. is still only half of all w.b. This leads to rather pessimistic conclusion that the effort and costs of running present water monitoring programs in Poland in about 50% of cases are likely wasted.

The presented analysis shows that in the case of water bodies assessed in *good* status, 22–52% of cases are characterized by PoM equal to 0.5 or higher, which means they likely are assessed falsely.

Moreover, a dozen or so percentage of results of ecological status assessments are accompanied with PoM higher than 0.5, which means that the status assessments are highly uncertain.

The most serious problem concerning the uncertainty of the w.b. status assessments seems to be the high values of PoMs assigned to the *good* and *moderate* classes, making the task of undertaking decisions on the corrective measures very ambiguous.

Examples of the riverine water status from the three provinces of Poland support a conclusion that the major problem of the country is eutrophication, which explains why, in majority of cases, the decisive water quality element for ecological status is either phytobenthos, i.e., diatom index, or concentration of nutrients.

The uncertainty resulting from introducing artificial disturbances to the outcomes of simple measurements (e.g., by randomly decreasing or increasing number of species individuals within different taxa) is smaller than the uncertainty arising from other types of errors (e.g., from wrong species identification). In that sense, the presented analysis, although rather pessimistic, can be considered too cautious.

Despite of quite a long period of time after introducing the WFD, monitoring data of priority substances in Poland are still scarce, in majority of w.b. thus not allowing to use them for reliable estimation of uncertainty measures of the overall w.b. status.

The next step, which still is to be made as to the analysis of probability of misclassification, is to find out a magnitude of PoM that would allow to accept or reject the assessed w.b. status at the assumed confidence level. Practical-related question is on frequency of measurements of water quality elements that can guarantee the desired, e.g., small enough, value of PoM*.*

Also, special analysis is needed to answer the question whether the two measures of uncertainty in given ecological status class—probability of misclassification and the standardized precision—could be used interchangeably. At present stage of research, they seem to be inconsistent.
